# Serum cholesterol and LDL-C in association with level of diastolic blood pressure in type 2 diabetic patients

**DOI:** 10.12861/jrip.2012.09

**Published:** 2012-01-01

**Authors:** Saeed Behradmanesh, Parto Nasri

**Affiliations:** ^1^Medical Plants Research Center, Shahrekord University of Medical Sciences, Shahrekord, Iran; ^2^School of Medicine, Isfahan University of Medical Sciences, Isfahan, Iran

**Keywords:** Cholesterol, Blood pressure, Dyslipidemia, Type 2 diabetes, Hypertension, Diabetic nephropathy, Lipids

## Abstract

**Introduction:** Elevated cholesterol and blood pressure are major risk factors for the development diabetic kidney disease. Possible interactions between these two parameters have not been studied in detail.

** Objectives:** This investigation aims to study the values of blood serum cholesterol, blood pressure and possible correlations between them, in a group of type 2 diabetic (T2D) patients.

** Patients and Methods:** A total of 60 patients with T2D were enrolled to the study. Venous blood samples were obtained in the fasting state for determinations of serum creatinine, lipids and hemoglobin A_1C_ (HbA_1c_).

** Results:** Of 60 participants, Mean of age was 57±8.3 years. Mean of systolic and diastolic blood pressure was 133±13 mmHg and 84±7.4 mmHg respectively. Mean of serum cholesterol and LDL-C was 182±34.5 mg/dl and 97.2 ±27.9 mg/dl respectively. In this study, a significant positive correlation of serum cholesterol with level of diastolic blood pressure (r= 0.286, p=0.030) was seen (adjusted for duration of diabetes and weight). Furthermore a significant positive correlation of serum LDL-C with level of diastolic blood pressure (r= 0.263, p= 0.044) was seen (adjusted for age).

**Conclusion:** We found a significant inverse correlation of serum cholesterol and LDL-C with level of diastolic blood pressure. This study showed the influence of serum lipids on the development of hypertension and further support the control of dyslipidemia, to prevent diabetic kidney disease.

**Implication for health policy/practice/research/medical:**

In a study on 60 type 2 diabetic (T2D) patients, a significant inverse correlation of serum cholesterol and LDL-C with level of diastolic blood pressure was observed. This study demonstrate the influence of serum lipids on the development of hypertension and further support the strict control of dyslipidemia, as a one of the factors aggravating hypertension and resultant nephropathy.

## Introduction

A number of risk factors for nephropathy are known to be present in diabetic patients, the most important being hyperlipidemia (1). Elevated cholesterol and elevated blood pressure are also the major risk factors for the development diabetic kidney disease (1,2). Indeed hypertension and diabetes are two common diseases and they affect the kidney as the major target organs. Also dyslipidemia and hypertension in patients with diabetes increase the risk of macrovascular complications (1-4).

Hypertension is a common condition in diabetes, affecting 20%-60% of patients with diabetes. The presence of high blood pressure and diabetes concomitantly accelerates the renal disease and decrease in renal function and the development of diabetic nephropathy (3-4). Possible interactions between the two parameters of lipids and blood pressure in diabetic patients have not been studied in detail.

## Objectives

This investigation aims to study the values of blood serum cholesterol, blood pressure and possible correlations between them, in a group of type 2 diabetic (T2D) patients.

## Patients and Methods

### Patients

A cross-sectional analytical study was carried out in 60 patients with T2D. They were under treatment of oral hypoglycemic agents or insulin therapy. Anthropometric measurements were obtained, including height, weight, Body mass index (BMI) was calculated in kilograms per square meter . Exclusion criteria were cigarette smoking, taking anti lipid drugs. Also patients who were under treatments of antihypertensive drugs or drugs which affects the lipid level or vitamin D metabolism were excluded from the study.

### Blood pressure measuring

The diagnosis of hypertension in people with diabetes is made if the mean of two readings on at least two clinic visits is ≥ 130/80 mmHg. The readings should be verified in the contra lateral arm. Resting systolic blood pressures and fifth phase diastolic blood pressures were measured three times while the subjects were seated, and the second and third measurements were averaged (4). Patients’ history, current medications, insulin doses, and tobacco use and family medical history were obtained.

### Laboratory methods

Venous blood samples were obtained in the fasting state for determinations of serum creatinine, lipids (total cholesterol (TC), low-density lipoprotein cholesterol (LDL-C), high-density lipoprotein cholesterol (HDL-C), and triglyceride measured ) and hemoglobin A_1c_ (HbA_1c_) (reference range 4–6%). Serum LDL-C was calculated by friedewald's formula (5). Creatinine clearance was evaluated from serum creatinine, age and body weight (6).

### Ethical issues

(1) The research followed the tenets of the Declaration of Helsinki; (2) informed consent was obtained; (3) the research was approved by ethical committee of Shahrekord University of Medical Sciences.

### Statistical analysis

Results were expressed as mean‏ ± SD and were considered as statistically significant when two-sided p<0.05. Independent-samples t-test was used for comparison variables between male and female. For correlations, partial correlation test was used.

## Results

Sixty patients were enrolled to the study. Of 60 participants, 56.7% were female. Mean of age was 57±8.3 years. Mean of diabetic duration was 9.2 ±4.9 years. Mean of HbA_1c_ was 7.4±1.0 %. Mean of serum creatinine was 0.98±0.22 mg/dl. Mean of serum cholesterol and LDL-C was 182±34.5 mg/dl and 97.2 ±27.9 mg/dl respectively. Mean of serum triglyceride and HDL-C was 221±99.1 mg/dl and 42.1 ±7.5 mg/dl respectively. Mean of systolic and diastolic blood pressure was 133±13 mmHg and 84±7.4 mmHg respectively. Table 1 summarized some of the data of patients. In this study, there was no significant difference of serum lipids and HbA_1c_ between males or females. Similarly, no significant difference of systolic or diastolic pressure between males and females was observed. In this study, a significant positive correlation of serum cholesterol with level of diastolic blood pressure (r= 0.286, p= 0.030) was seen (adjusted for duration of diabetes and weight of the patients). Furthermore, a significant positive correlation of serum LDL-C with level of diastolic blood pressure (r= 0.263, p= 0.044) was seen (adjusted for age of the patients).There was not significant association between level of systolic pressure with serum cholesterol or LDL-C values (p> 0.05).There was not significant association between serum triglyceride or HDL-C serum values with levels of systolic or diastolic pressure (p> 0.5).

**Table 1:** The results of variables in type 2 diabetic patients

**Variable**	**Min**	**Max**	**Mean ± SD**
BMI (kg/m^2^)	20.06	41.4	29.9 ± 4
Diabetic duration (years)	3	23	9.2 ± 4.9
Systolic BP (mmHg)	110	170	133 ± 13
Diastolic BP (mmHg)	70	100	84 ± 7.4
Serum creatinine (mg/dl)	0.7	1.9	0.98 ± 0.22
Serum calcium (mg/dl)	8.2	10	9.0±0.4
Serum cholesterol (mg/dl)	120	280	182±34.5
HbA_1_c (%)	4.8	9.5	7.4 ± 1.07
BP, Blood pressure.			

## Discussion

Hypertension is a disease with multi-factorial etiologies. The prevalence of hypertension in patients with T2DM is known to be 1.5–3 times higher than in the age-matched non diabetic population (1-4).Various hypotheses were proposed about the possible mechanisms underlying hypertension including disturbances in serum electrolytes (4-8), however least studies published regarding the influence of lipids on blood pressure in diabetic patients. In this study, we found a significant positive correlation of serum cholesterol and LDL-C with levels of diastolic blood pressure .There was not significant association between levels of systolic pressure with serum cholesterol or LDL-C values .There was not significant association between serum triglyceride and HDL-C values with level of systolic or diastolic pressure. In the study of Thakur *et al*. on lipid profiles of 3,182 uncomplicated non-diabetic patients, an abnormal total cholesterol-HDL ratio as the most common variety of dyslipidemia in uncomplicated hypertension was observed (9). Schaars *et al*, gathered data of 895 randomly selected diabetic patients of 95 general practitioners, showed that 73%, were hypertensive in the last year (10). Lautsch *et al*. studied a total of 7496 outpatients with arterial hypertension in Austria. Men and women had similar systolic and diastolic blood pressure and similar non-HDL- C values. They found a correlation of non-HDL- C and blood pressure in hypertensive men of all age groups (11). Recently Martin *et al*. found a significant relationship between measures of morning blood pressure surge and LDL-cholesterol (12). It is possible that a biological model of impaired endothelial function or of an up regulation of the AT1 receptor by low density lipoprotein cholesterol (13), leading to an increase in diastolic blood pressure.

## Conclusion

A significant inverse correlation of serum cholesterol and LDL-C with level of diastolic blood pressure was observed in your study. This study demonstrate the influence of serum lipids on the development of hypertension and further support the strict control of dyslipidemia, as a one of the factors aggravating HTN and resultant nephropathy.

## Authors’ contributions

SB conducted the research. PN wrote the manuscript.

## Conflict of interests

The authors declared no competing interests.

## Ethical considerations

Ethical issues (including plagiarism, informed consent, misconduct, double publication and redundancy) have been completely observed by the authors.

## Funding/Support

This study was funded by Shahrekord University of Medical Sciences (grant# 898).
